# Iron Supplementation in Pregnancy and Risk of Gestational Diabetes: A Narrative Review

**DOI:** 10.3390/nu14224791

**Published:** 2022-11-12

**Authors:** Clive J. Petry

**Affiliations:** Medical Research Laboratories, Wellcome-MRC Institute of Metabolic Science, University of Cambridge, Cambridge CB2 0QQ, UK; cjp1002@cam.ac.uk; Tel.: +44-(0)1223-274218

**Keywords:** iron, diabetes, pregnancy, insulin secretion, insulin resistance, micronutrients, oxidative stress, dietary supplementation, ferritin

## Abstract

Pregnant women frequently supplement their diets with iron to treat any cryptic anemia, on the assumption that if anemia is not present, there will be no negative consequences. However, in women who are already iron-replete, it has been suggested that this can lead to iron overload and an increased risk of certain pregnancy complications. One such complication is gestational diabetes. Fourteen clinical trials, case–control or cohort studies (found using Pubmed/Scopus/Web of Science) have investigated links between iron supplementation in pregnancy and risk of gestational diabetes, several of them finding significant associations with increased risk. Potential mechanisms include increased oxidative stress leading to insulin resistance and inadequate compensatory insulin secretion. Current evidence suggests that dietary supplementation with iron in pregnancy may increase a pregnant woman’s chance of developing gestational diabetes, although available evidence is somewhat contradictory, and the magnitude of any increased risk appears relatively small. Meta-analyses have suggested the presence of significant heterogeneity in results between studies, urging a degree of caution in interpreting these results. It is currently suggested that advice to pregnant women about whether to supplement their diets with iron or not should consider both their current iron status and their other established risk factors for gestational diabetes.

## 1. Introduction

Iron is an essential micronutrient for a number of different processes in the body, including the handling of oxygen for transport and storage (as part of hemoglobin in red blood cells and myoglobin in muscle cells), ATP production and energy release, DNA biosynthesis, the synthesis of collagen and neurotransmitters, and immune function [1]. The body’s iron requirements increase considerably during pregnancy (from about 0.8 mg/d absorbed iron in the first trimester to 7.5 mg/d in the third trimester, with an average requirement across pregnancy of around 4.4 mg/d [2]) to support fetal and placental growth and the enlargement of maternal blood volume [3]. Iron absorption in the gut is thought to rise with increasing gestation [2] to help mediate this. The large increase in the body’s requirement for iron makes pregnancy a stage of life that is particularly prone to iron deficiency, in populations from both developed and developing countries. Given that iron deficiency (anemia) in pregnancy can lead to an increased risk of preterm birth [4] and of having a low-birth-weight/intrauterine-growth-restricted baby, the majority of countries worldwide recommend that iron supplements are used in pregnancy to augment dietary iron intakes [3,5]. Even some of the countries that do not recommend it universally, such as the U.K., suggest that iron supplements are used by pregnant women diagnosed with either iron deficiency or iron deficiency anemia [6,7].

Iron deficiency is the most common micronutrient deficiency in the world [8] and is thought to cause a number of short- and long-term complications [9]. It also appears to lower the risk of the pregnant woman developing gestational diabetes (GDM) (diabetes that is first detected in pregnancy) when manifesting as iron deficiency anemia [10]. In contrast, iron overload is thought to raise the risks of pregnancy complications such as pre-eclampsia and possibly GDM [11], especially where women who are already replete with iron supplement their dietary iron intakes. Initial evidence for this comes from genetic studies related to the regulation of body iron stores. One study reported raised iron stores and increased GDM incidence in women with heterozygous hemoglobinopathies [12]. In another study, the allele frequency of a hereditary hemochromatosis mutation (C282Y) was raised in women with GDM [13]. Similarly, risk alleles of single-nucleotide polymorphisms in the *TMPRSS6* gene (which codes for the protein matriptase-2) were found to be associated with serum iron concentrations and transferrin saturation, as well as with GDM in another study [14]. Finally, the haptoglobin phenotype, which relates to allelic variants in the haptoglobin gene, has also been reported to be associated with the development of GDM [15]. Independent of genetics, increased iron stores (whether assessed by blood hemoglobin, serum iron, ferritin, transferrin (saturation), or soluble transferrin receptor) have rather consistently been associated with an increased risk of GDM (see [16,17,18,19,20,21] for systematic reviews of the primary literature and meta-analyses of the subject). Causes of increased stored iron can be genetic but it can also be acquired through such factors as frequent blood transfusions, injected iron preparations or high iron intakes. Iron intakes can be dietary, two thirds of which tends to be in the form of heme iron and the rest in the form of inorganic iron in developed countries [22], or supplemental also as inorganic iron, in the form of salts such as ferrous gluconate, fumarate, succinate, or sulfate. High dietary heme iron intake has been associated with the development of GDM in some studies [19,21,23,24,25,26]. However, the question remains as to whether the same is true for supplemental iron intake in pregnancy. This review, therefore, looks at evidence for this link between supplemental iron intake during pregnancy and the development of GDM.

## 2. Methods

Three databases (Pubmed, Scopus and Web of Science) were used to search for publications to include in this review (Table 1). Two searches of each database were performed to identify manuscripts that could be included in the Introduction and the part of the Discussion section of the review related to mechanism (where not all manuscripts that were identified were cited in this review), and also those specifically about iron supplementation in pregnancy (i.e., between conception and birth) and GDM to be included in the main Discussion section of the review (where all the manuscripts that were identified were cited in this review). The only language limitation put on publications for inclusion was that the abstract was written in English (i.e., there was no language limitation put on what language the other (non-abstract) parts of the manuscript were written in). Most of the manuscripts included in this review from the databases were primary research publications (randomized control trials, case–control studies, or cohort studies). However, relevant secondary research publications (including systematic reviews and meta-analyses, and narrative and scoping reviews) were also included, and their citations were checked to identify any further relevant primary research manuscripts. The list of publications was then narrowed by the author of this review into those suitable for its main section, categorizing them according to the type of study.

## 3. Discussion

### 3.1. Studies Seeking Associations between Iron Supplementation in Pregnancy and GDM

#### 3.1.1. Randomized Controlled Trials

Three randomized controlled trials, considered to potentially provide the most reliable evidence of the effectiveness of interventions due to a lower risk of bias, have been performed to assess associations between iron supplementation (or differences in the dose of iron supplementation) and the development of GDM [27,28] or a composite variable made up of factors related to GDM [29] (Table 2). None of the trials have provided evidence of a link between iron supplementation in pregnancy and the development of GDM, although in one of the trials, the dose of iron used was very low, as was the recorded background incidence of GDM [28].

#### 3.1.2. Case–Control Studies

Four case–control studies have sought associations between iron supplementation in pregnancy and GDM development [30,31,32,33] (Table 3). They produced inconsistent results as two studies found a significant association [31,32] and two did not [30,33]. A further study not included in Table 3 [34] retrospectively looked at 52 women with GDM and 50 women without GDM, all of whom supplemented their diets with 40 mg iron/day from around week 16 of pregnancy. At weeks 24~28, those women that developed GDM had higher ferritin and hemoglobin levels, although baseline values (~week 16 of pregnancy) were not presented. Although these results could have been confounded by differences in dietary iron intakes and in baseline iron characteristics, it is possible that as well as iron supplementation being a factor in the development of GDM, its absorption and storage may also be factors [35]. In contrast, Gungor et al. [36] studied 56 women with GDM and 56 pregnant women without GDM, the diet of all of whom was supplemented with a multivitamin preparation containing 29 mg iron/tablet (one tablet taken per day) after an ‘initial’ hospital visit until delivery and found no difference in serum ferritin concentrations or blood hemoglobin levels at 28–30 weeks of pregnancy. The dose of supplemental iron may therefore also be a factor.

#### 3.1.3. Cohort Studies

Seven prospective cohort studies investigated associations between iron supplementation in pregnancy and the development of GDM (Table 4). Again, inconsistencies were found in the results with two studies failing to find significant associations [26,37] and five studies reporting significant positive associations [38,39,40,41,42]. Within studies, there were other inconsistencies as one of the studies that failed to find a significant association between iron supplementation in pregnancy and GDM did find a significant association with pre-pregnancy iron supplementation [37], and three studies that did find a significant association also reported a lack of association at different time points of supplementation or when using lower doses of iron [39,40,41].

#### 3.1.4. Systematic Reviews with Meta-Analyses

Inconsistencies in results from the different primary research studies relating to iron supplementation in pregnancy and the risk of developing GDM make it difficult to draw meaningful conclusions. Further insight has been gained from systematic reviews and meta-analyses [16,19,23,43] (Table 5), which were performed in an effort to obtain a more precise estimate of the size of any modifying effect of iron supplementation in pregnancy. However, the different meta-analyses produced varying outcomes as they were based on different meta-analysis models and a different number of studies, due to new primary research studies being published after the meta-analyses were performed, and differences in a priori inclusion and exclusion criteria. Three of them failed to find any significant association between iron supplementation in pregnancy and the development of GDM [16,19,23]. In contrast, one meta-analysis found a significant association with an odds ratio of 1.3, but with significant heterogeneity between results from different studies [43].

### 3.2. Potential Mechanisms of How Iron Supplementation in Pregnancy Could Affect GDM Risk

Like most forms of non-autoimmune diabetes, GDM is thought to result from a combination of insulin resistance and inadequate insulin secretion to compensate for the degree of insulin resistance. If iron supplementation in pregnancy in women who are iron-replete was able to increase the risk of them developing GDM [44], you would therefore expect it to do so by affecting insulin secretion and/or insulin sensitivity. Through its ability to react with oxygen, iron can increase the production of reactive oxygen species [45], which could damage proteins, lipids and nucleic acids unless deactivated by defense mechanisms such as antioxidant enzymes and buffering proteins. Indeed, iron supplementation in pregnancy has been reported to increase rates of reactive-oxygen-species-related lipid peroxidation, described as being predictive of adverse effects on both the mother and fetus [46]. Pancreatic β-cells, which produce and secrete insulin, both processes of which are exquisitely iron-dependent, appear particularly susceptible to damage from reactive oxygen species (reviewed in [47]). This is, in part, due to their low expression of antioxidant enzymes such as superoxide dismutase, glutathione peroxidase, and catalase. They also appear prone to the accretion of iron [48]. Iron overload, especially in iron-replete women, could intensify the buildup of reactive oxygen species and oxidative damage, ultimately leading to the apoptosis of pancreatic β-cells and a resulting reduction in insulin secretion [49].

In addition to effects on insulin secretion, iron may also promote insulin resistance in pregnancy through increased lipid peroxidation reducing glucose utilization in muscle and increasing hepatic gluconeogenesis [50]. Consistent with this, one study reported that increased insulin resistance stratified by serum ferritin concentrations (which can be modified by iron supplementation in pregnancy [51]) was associated with impaired glucose tolerance and GDM risk [44].

## 4. Conclusions

**Figure 1:**
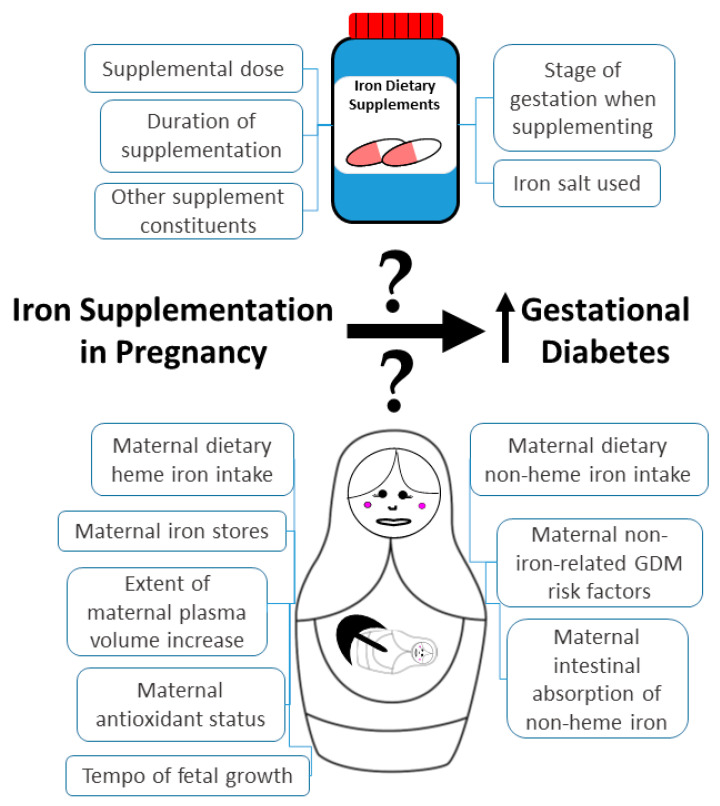
Some of the possible causes of heterogeneity in studies attempting to test whether iron supplementation in pregnancy is associated with an increased risk of developing GDM.

There is conflicting evidence in the literature as to when iron supplementation in pregnancy is able to increase the risk of GDM development, if indeed it is. Even the recent (and most thorough) systematic review and meta-analysis that demonstrated a significant positive association between iron supplementation in pregnancy and GDM risk [43] did so with highly significant heterogeneity. Its exclusion criteria also meant that randomized controlled trials were not included in the meta-analysis. All of these [27,28,29] have thus far failed to show an association between iron supplementation in pregnancy and GDM risk, although they all have limitations which may have contributed to their inabilities to find such associations (Table 2 and Table 5). The heterogeneity in results of studies discovered in the meta-analysis [44] could relate to factors such as between-individual and between-study differences in participant baseline iron stores, dietary (heme and non-heme) iron intakes, doses of iron supplementation, duration of supplementation, gestational stage when supplementation took place, and relative amounts of non-heme iron that is absorbed from the intestine. Non-iron factors that may affect heterogeneity include the extent of pregnancy-related increases in plasma volume, the mother’s antioxidant status, the presence of other GDM risk factors in the mother, and the tempo of fetal growth. All these factors make it more difficult to draw conclusions about the role of iron supplementation in GDM risk (Figure 1). However, there is considerable evidence from the literature linking iron overload with GDM development [23,52] and a potential mechanism, making it plausible that iron supplementation in pregnancy contributes to risk, especially in iron-replete women.

More convincing evidence would need to come from randomized controlled trials, but these are likely to require some degree of control of the sources of heterogeneity to maximize the chance of gaining convincing findings. Until such studies are performed, all that we can say is that the current literature suggests that iron supplementation in pregnancy *may* increase the risk of developing GDM, especially in iron-replete women [11]. However, the increased risk appears relatively small and may only be of significant consequence for those iron-replete women with other risk factors for GDM. Given the established negative effects of iron deficiency (anemia) in pregnancy [3] it is perhaps too early to counsel against iron supplementation in pregnancy, especially in women without other risk factors for GDM. However, this may happen in the future, especially in women whose iron status is screened and confirmed to be adequate in pregnancy.

## Figures and Tables

**Table 1:** The search criteria and databases used to find manuscripts for this review.

Date of Search	Database	Years Searched	Search Terms	Strings of Terms	# Hits
1 September 2022	Pubmed	1966-08/2022	iron, gestational diabetes	iron AND gestational diabetes	238
1 September 2022	Pubmed	1966-08/2022	iron supplementation, gestational diabetes	iron supplement* AND gestational diabetes	85
1 September 2022	Scopus	1960-08/2022	iron, gestational diabetes	iron AND gestational AND diabetes	452
1 September 2022	Scopus	1960-08/2022	iron supplementation, gestational diabetes	iron AND supplement* AND gestational AND diabetes	163
1 September 2022	Web of Science	1900-08/2022	iron, gestational diabetes	iron gestational diabetes	352
1 September 2022	Web of Science	1900-08/2022	iron supplementation, gestational diabetes	iron supplement* gestational diabetes	133

* signifies a wildcard. The databases used were: Pubmed (URL pubmed.gov (accessed on 1 September 2022)), Scopus (URL https://www.elsevier.com/en-gb/solutions/scopus (accessed on 1 September 2022)), and Web of Science (URL https://clarivate.com/webofsciencegroup/solutions/web-of-science/ (accessed on 1 September 2022)).

**Table 2:** Randomized controlled trials that sought associations between iron supplementation in pregnancy and GDM.

First Author, Year of Publication	Details	Key Results	Comments
Chan, 2009 [27]	Women with baseline hemoglobin concentrations of between 8 and 14 g/dL attending Hong Kong University Queen Mary Hospital, Hong Kong were randomly allocated to receive 60 mg of elemental iron supplement (as 300 mg ferrous sulphate) daily (*n* = 565) or placebo (*n* = 599) from their pregnancy hospital booking visit. There was no difference in baseline hemoglobin or ferritin levels, or between the ratio of ferritin to transferrin between the groups. Iron supplementation (or placebo) was administered for a further 16 weeks.	In the comparison of those that supplemented with iron against those that took the placebo, there was no significant difference in the incidence of GDM at 28 weeks’ gestation (~11% in both groups; odds ratio for GDM 1.04 (0.7–1.53), *p* = 0.9).	There was a relatively low general compliance and involvement in the follow-up. ~40% were lost to follow-up. Maternal ferritin (*p* = 0.003) and hemoglobin (*p* < 0.001) concentrations were higher in the iron supplement group at delivery. Offspring birth weight was increased ~100 g in iron-supplemented pregnancies (*p* = 0.001).
Ouladsahebmadarek, 2011 [28]	A double-blind-randomized clinical trial of 960 women attending Al Zahra Hospital in Isfahan, Iran, with singleton pregnancies in the first trimester who had not supplemented with iron in the preceding month and who had hemoglobin concentrations >12 g/dL. The supplemental group (*n* = 480) took 30 mg elemental iron/d plus an unspecified multivitamin preparation from 13 weeks of pregnancy until delivery. The control group (*n* = 480) took a placebo plus the multivitamin preparation.	In the comparison of those that supplemented with iron against those that took placebo, there was no significant difference in the incidence of GDM (0.5% vs. 0.8%, respectively; relative risk 0.67 (0.11–3.97), *p* = 0.7).	85% of those that supplemented with iron and 78% of those who took placebo completed the course. GDM was recorded by a questionnaire, not from hospital notes or by performing oral glucose tolerance tests.
Kinnunen, 2016 [29]	Women from Tampere and five neighboring municipalities in Southern Finland were randomly allocated to receive 100 mg iron supplement daily (*n* = 1336; “routine” group) throughout pregnancy (regardless of their hemoglobin level) or no supplementation (*n* = 1358) (unless they were diagnosed with anemia (“selective” group), in which case they were supplemented with 100 mg/d (as two doses of 50 mg iron) just until their hemoglobin level increased to 11 g/dL).	In the comparison of those that routinely supplemented with iron against those that either did not or only did so selectively, there was no difference in the incidence of a composite variable related to glucose intolerance (GDM, glycosuria, and large-for-gestational-age baby): 11.0% v. 13.0% (*p* = 0.1), respectively.	This was a reanalysis of an original trial where GDM was not a planned outcome and therefore was not assessed systematically in all participants. Instead, GDM, glycosuria, and/or large-for-gestational-age records were abstracted from hospital records and combined into a composite variable. Few participants were overweight or obese.

**Table 3:** Case–control studies that sought associations between iron supplementation in pregnancy and GDM.

First Author, Year of Publication	Details	Key Results	Comments
Palma, 2008 [30]	A retrospective study of 1256 pregnant women without anemia (322 who delivered a low-birth-weight baby; 934 who delivered non-low birth-weight-baby at term), attending the Marqués de Valdecilla University Hospital, Santander, Spain. Information about iron and other supplements was obtained from personal interviews and prenatal care records. A total of 91.0% of the women supplemented with iron, mainly with a daily dose of 80 mg ferrous sulfate as a single supplement. No details of baseline iron statuses were presented, although none of the women were anemic. Three sources of data for GDM were used: personal interviews (carried out within three days after delivery), clinical charts, and prenatal care records.	In the comparison of those that supplemented with iron against those that did not, no association was found between iron supplementation and GDM (4.6% of women who supplemented with iron developed GDM vs. 7.1% of women who did not supplement with iron developed GDM; risk ratio 0.65 (0.32–1.34), *p* = 0.2).	A woman was considered to have supplemented with iron if they took it for at least one week in pregnancy. No account of iron supplemental dose or duration (other than the above) was taken. Maternal iron supplementation was associated with a lower risk of low birth weight (odds ratio 0.58 (0.34–0.98), *p* < 0.05).
Bo, 2009 [31]	A study of 500 consecutive pregnant women with GDM and 500 normoglycemic women attending the Unit of Obstetrician and Gynecology of the University of Turin, Italy. Iron supplementation data were collected by interviewing. A woman was considered to have supplemented with iron if she did so for at least 2 weeks. Most (95.5%) of the 212 women that supplemented with iron (and no other micronutrients) in mid-pregnancy did so with a daily dose of 525 mg ferrous sulfate (equivalent to 105 mg elemental iron). No details were presented about baseline iron statuses or anemia.	In the comparison of those that supplemented with iron against those that did not, self-reported iron supplementation in pregnancy was associated with a higher prevalence of GDM (70.8% v. 44.4% (*p* < 0.001), respectively; unadjusted odds ratio 3.03 (2.18–4.20), *p* < 0.001; odds ratio adjusted for confounders 3.36 (1.50–7.53), *p* = 0.003). The duration of iron supplementation ranged from 2 to 10 weeks (with a median of 5 weeks). Only one participant was supplementing with iron prior to conception (and sensitivity analyses removing them showed no significant difference in results).	Iron supplementation in pregnancy was also significantly positively associated with increased insulin resistance and hyperglycemia, and the prevalence of the metabolic syndrome.
Jirakittidul, 2018 [32]	A retrospective study of pregnant women attending Vajira Hospital, Bangkok, Thailand for routine antenatal care. The early iron supplementation group (*n* = 966) was non-anemic women who started taking oral iron supplements (200 mg of ferrous fumarate; classified from hospital records) prior to 16 completed weeks of gestation, while the “control” group (*n* = 969) was those non-anemic women who started supplementing with iron later on in pregnancy. There was no difference in baseline hemoglobin levels between the groups.	In the comparison of those that supplemented with iron early against those that supplemented late, those that supplemented early had a higher prevalence of GDM (9.7% vs. 5.6%, respectively; risk ratio 1.83 (1.29–2.59), *p* < 0.001).	In this study, the control group consisted of women who supplemented their diets with iron, but only after 16 weeks’ gestation. There was no group where the women did not supplement their diets with iron.
Liu, 2018 [33]	A retrospective study of 259 women with singleton pregnancies and hemoglobin levels between 8–14 g/dL attending The People’s Hospital of Yan’an, and Affiliated Hospital of Yan’an University, Yan’an, China. The supplementary group (*n* = 135) took 300 mg iron/d from prior to 16 weeks’ gestation until delivery. The control group did not supplement their diets with iron.	In the comparison of those that supplemented with iron against those that did not, there was no significant difference in the prevalence of GDM (7.4% vs. 7.3%, respectively; risk ratio 1.02 (0.43–2.43), *p* = 1.0).	There were no reports of either the method of participant recruitment or how iron supplemental data were captured. Iron supplementation was associated with increased maternal hemoglobin levels at delivery and increased offspring birth weight.

**Table 4:** Cohort studies that sought associations between iron supplementation in pregnancy and GDM.

First Author, Year of Publication	Details	Key Results	Comments
Bowers, 2011 [26]	13,475 pregnancies (which took place from 1991 to 2001) from the Nurses’ Health Study II (a prospective study of female nurses in the U.S.A., aged 22–44 at recruitment in 1989). No information was presented about baseline iron statuses.	In the comparison of no iron supplementation in pregnancy against other quintiles of iron supplementation dose in pregnancy, no significant associations were observed with GDM risk (age-adjusted risk ratios 0.80~0.99 (0.62~1.04), p_trend = 0.6) (multivariate-adjusted risk ratios 0.86~1.04 (0.68~1.28), p_trend = 0.97).	For those women that supplemented their diets with iron, no restriction was placed on when iron supplementation started, or on its duration. It was not recorded whether the supplemental iron was part of a multiple micronutrient preparation or not. Analysis was of supplemental iron groups (0, ~5.1, ~15, ~30, and ~60 mg iron/d). GDM was self-reported.
Zhu, 2019 [37]	3289 pregnant women from the Ma’anshan Birth Cohort, conducted in Ma’anshan city of Anhui province in China. No information was presented about baseline iron statuses.	Pre-pregnancy iron supplementation was associated with an increased risk of GDM (supplemented 18.4% vs. non-supplemented 12.6%; risk ratio 1.45 (1.06–1.97), *p* = 0.02). No significant associations between iron supplement use and risk of GDM in either first trimester (14.5% vs. 12.6%, respectively; risk ratio 1.16 (0.95–1.41), *p* = 0.1) or second trimester (14.5% vs. 13.0%, respectively; risk ratio 1.12 (0.87–1.43), *p* = 0.4) were observed, nor was there an interaction with iron supplementation in the apparently U-shaped association between hemoglobin level and GDM incidence.	Iron supplementation was self-reported by questionnaire. Both sole iron supplements and multivitamin/mineral supplements that contain iron were included as “iron supplement use.” No account was taken of dose or total duration of iron supplementation. Iron supplements were consumed by 5.9% before pregnancy, 23.6% in the first trimester, and 13.2% in the second trimester.
Hao, 2020 [38]	Purposive sampling from 807 pregnancies from Chengdu City hospital in China. No information was presented about baseline iron statuses.	There was a positive association between the iron supplement dose and the occurrence of GDM in 739 women during the second trimester of pregnancy (odds ratio 1. 06 (1. 02–1.10), *p* < 0.05). Compared with the lower iron supplemental intake (<60 mg iron/d) women in mid-pregnancy, the odds ratio for GDM was 1.41 (1.02–1. 94) (*p* < 0.05) in the higher iron supplemental intake (≥60 mg iron/d) women.	There was no group analyzed where none of the women supplemented with iron. A total of 5% of the women supplemented with iron in the first trimester of pregnancy and 67.9% in the second trimester (~32% supplementing with ≥60 mg iron/d).
Si, 2021 [39]	1118 pregnancies from the Zhoushan Pregnant Women Cohort based in Zhoushan Maternal and Child Care Hospital, China. Iron was supplemented in the form of iron polysaccharide complex capsules or ferrous succinate tablets of unreported dose. No information was presented about baseline iron statuses.	223 of the women developed GDM, whereas 905 did not. First trimester iron supplementation was not associated with GDM risk (risk ratio 1.21 (0.93–1.55), *p* = 0.2). However, iron supplementation in the second trimester in non-anemic women was associated with increased GDM risk (risk ratio 1.34 (1.06–1.70), *p* = 0.01).	Although an account was taken of the trimester in which iron was supplemented, no account was taken of the dose or the total duration of supplementation.
Zhang, 2021 [40]	2117 pregnancies from the Tongji Maternal and Child Health Cohort from Wuhan, China: the same cohort but different analysis as [41]. Iron supplements that were taken by the participants were most commonly in the form of ferrous fumarate (19.8 or 60 mg/d) or ferrous glycinate (5 mg/d). Iron statuses at baseline (~16 weeks gestation) were collected in terms of plasma ferritin concentrations.	Supplemental iron at a dose of ≥60 mg/d during the second trimester of pregnancy was associated with an increased risk of GDM compared with non-users (14.0% vs. 8.9%, respectively; unadjusted risk ratio 1.56 (1.16–2.11), *p* = 0.003; adjusted risk ratio 1.37 (1.02–1.84), *p* = 0.04). Participants with medium plasma ferritin concentrations and high supplemental iron intake (supplemental iron ≥60 mg/d) were associated with an increased risk of GDM (14.7% vs. 8.4% in women who did not supplement), similar to the participants within the highest quartile of plasma ferritin concentrations (≥90.0 ng/mL) in early pregnancy (14.3%). This was possibly due to higher intestinal iron absorption in those without the highest ferritin concentrations. Supplementing with lower doses of iron or for a shorter duration was not significantly associated with GDM risk (*p* > 0.05).	High-dose iron supplementation was defined as elemental iron ≥60 mg/d on ≥5 days/wk for at least 4 wk. Non-users were those who reported no intake of iron-containing supplements.
Zhang, 2021 [41]	5101 pregnancies from the Tongji Maternal and Child Health Cohort from Wuhan, China: the same cohort but different analysis as [40]. Iron statuses at baseline (~16 weeks gestation) were collected in terms of hemoglobin concentrations.	The Incidence of GDM was significantly higher in women who supplemented with ≥30 mg iron/d for more than 3 months in pregnancy than in those that did not supplement with iron (15.2% vs. 8.9%, *p* < 0.001). The unadjusted risk ratio for GDM was 1.70 (1.40–2.07). After adjustment for confounders, the risk ratio was 1.55 (1.27–1.88). Supplementing with lower doses or for a shorter duration was not significantly associated with GDM risk (all *p* > 0.05).	Iron supplements included both iron-only supplements and multiple micronutrient preparations that contained iron. Those considered to supplement with iron were women who supplemented with iron more than 5 times per week on average. Iron supplementation (any dose) was associated with higher hemoglobin levels at delivery.
Petry, 2021 [42]	677–868 pregnancies from the Cambridge Baby Growth Study recruited from the Rosie Maternity Hospital, Cambridge, U.K.	Around 61% of the pregnant women self-reported supplementing their diets with iron. In the comparison of those women that supplemented with iron against those that did not, maternal iron supplementation in pregnancy was associated with increased GDM risk (risk ratio 1.67 (1.01–2.77), *p* = 0.048, *n* = 677).	No details of baseline iron statuses are presented, although ~3% of the participants reported having anemia in pregnancy. For those women that supplemented their diets with iron, no restriction was placed on when iron supplementation started, or its dose or duration. The median duration of supplementation was ~34–36 weeks (starting when pregnancy was confirmed). Most women that supplemented (~90%) did so using multiple micronutrient preparations at a dose of ~28-34 mg iron/d. Associations with one of the secondary phenotypes (offspring subscapular skinfold thickness at birth) was still evident when data from women who used multiple micronutrient preparations were excluded.

**Table 5:** Meta-analyses that sought associations between iron supplementation in pregnancy and GDM.

First Author, Year of Publication	Details	Key Results	Comments
Khambalia, 2015 [16]	Included just two randomized controlled trials, neither of which found associations between iron supplementation and GDM.	There was no significant association between iron supplementation in pregnancy and risk of GDM.	Both trials assessed iron supplement use in early pregnancy and found no association with risk of GDM. The Chan et al. trial scored highly (92%) in the quality assessment [27] following careful design. The other trial [28] scored poorly in the quality assessment (58%). It collected GDM data from a questionnaire rather than from hospital notes or oral glucose tolerance tests, had considerable loss to follow-up, and did not report on the methods of randomization or blinding used.
Zhao, 2017 [19]	Included four published studies: one case–control study, one cohort study, and two randomized controlled trials.	There was no significant association between iron supplementation in pregnancy and GDM in the cohort studies (risk ratio 1.75 (0.56–5.47); I^2^ = 86.8%), nor was there one for the randomized control trials (risk ratio 0.88 (0.72–1.07); I^2^ = 0%).	No overall pooled result for associations between iron supplementation in pregnancy and risk of GDM was presented.
Kataria, 2018 [23]	Included four published studies: two case–control studies, one cohort study, and one randomized controlled trial.	Supplemental iron intake was not associated with risk of GDM (unadjusted odds ratio 1.20 (0.63–2.29), odds ratio adjusted for confounders 1.09 (0.73–1.63)) with significant heterogeneity (unadjusted I^2^ = 93.7%, *p* = 2.26 × 10^−10^; adjusted I^2^ = 82.7%, *p* = 0.003).	There was no evidence of publication bias.
Moradi, 2022 [43]	Included 10 published studies: 2 case–control, 1 retrospective, and 7 prospective cohort designs.	There was a significant positive association between iron supplementation in pregnancy and GDM (odds ratio 1.30 (1.10–1.55), *p* = 0.002) with significant heterogeneity (I^2^ = 87.6%, *p* < 0.0001). Subgroup analyses revealed a significant association between iron supplementation in pregnancy and risk of GDM in cohort studies (odds ratio 1.23 (1.06–1.43), *p* = 0.005) with significant heterogeneity (I^2^ = 82.6%, *p* < 0.0001). This association was not observed in case–control studies (odds ratio 1.45 (0.32–6.65), *p* = 0.6; heterogeneity I^2^ = 92.4%, *p* < 0.0001).	Randomized controlled trials were not included in the meta-analysis as they did not fit the inclusion criteria. Further subgroup analyses revealed a significant association in high-quality studies (odds ratio 1.32 (1.10–1.58), *p* = 0.002) (I^2^ = 89.2%, *p* < 0.0001), but not in fair-quality studies (odds ratio 1.08 (0.43–2.76), *p* = 0.9) (I^2^ = 75.7%, *p* = 0.04). There was no evidence of publication bias.

## Data Availability

Not applicable.
